# Mr. Pearson, on Vaccination in China

**Published:** 1808-12

**Authors:** W. G. Maton

**Affiliations:** Spring Gardens


To Dr. BRADLEY.

Dear Sir,

I Transmit to you the enclosed, which has recently been received from Canton, by Sir George Thomas Staunton, Bart. and which I will beg the favour of you to insert in the Medical and Physical Journal, as I think it cannot but prove interesting to your readers.

I am, &c.

W. G. MATON.

*Spring Gardens, Sept.* 26, 1808.

---

IN the 10th Volume of the Medical Journal, I observed, under the head of Medical Intelligence, an article which contained a short account of the introduction of the Vaccine Inoculation into this empire. As this has been deemed by you of sufficient interest to merit insertion, I think it not improbable that a sketch of such farther progress as has been made here in the practice, may be acceptable to some of your readers. On its first introduction, I communicated a detailed account of the success of the experiment to Dr. John Hunter, leaving it to him whether any part of it should be made public. I infer that he has not deemed it of sufficient utility from the consideration that at this period of the discovery, and after so much inquiry and disquisition on the subject from every quarter, my communication could afford no result profitable to science, however gratifying it might be as a piece of intelligence to all who feel an interest in the progress of so singular and beneficial a discovery, more especially to those who have a professional concern in what promises to lessen so materially the sum of human misery.

The article in your Journal which I allude to, gives an abstract of the introduction, which was by means of virus brought on live subjects from Manilla by Mr. Huet, a respectable spectable Portuguese merchant of the latter settlement; the practice was favoured by the breaking out of the small-pox soon after in Canton, and the opinion then held as to the most advisable mode of making it known, was to vaccinate as many as possible; not fully aware of the characteristical apathy of the Chinese, and thinking that such a benefit to be appreciated, required only to be known. Several thousands were in the course of twelve months vaccinated, nor have I yet heard an imputation which could bear being traced, against the success of the practice; this is the more extraordinary, as in completion of my views on the subject, I instructed several Chinese as well as I was able, in the mode of conducting it, and they practised it extensively, as well at a distance from my inspection as under it. Whenever the small-pox ceased to be epidemic, the evil and the resource were equally forgotten, and it was with great difficulty that subjects to keep up a supply of the fluid were procurable. At last, in December, 1800, it failed in two at one time; and I found a supply of virus procured from Macao to produce the spurious pustule only; I therefore relinquished the practice for a time, trusting to a fresh supply from India or Manilla. Towards the spring of 1807, the small-pox again made its appearance, not raging so violently as before among the natives, but proving dangerous or fatal to such strangers as were unfortunate enough to be attacked with the disease. I was under the necessity of inoculating five persons with variolous matter; they were adults, but not Chinese, and I trust every due observance was had, both in precaution and treatment, but the disease proved in them all severe and dangerous, and in one case fatal. There was also an opportunity of applying the tests of variolation and exposure, to several who had been vaccinated from one to five years before, without small-pox being produced.

We remained without the vaccine until the month of July, 1807, when it was again brought upon the live subject in a Portuguese ship from Manilla, by the direction of Lieut. Ariaga; and in consequence of his official interference as chief magistrate of Macao, and by his attention, I received a fresh stock, and was enabled to recommence the practice among the Chinese. Since returning to Canton, it has been kept up there, but under an alteration of the plan, as I now do not vaccinate more than ten or twelve at a time, wishing that those who want to be benefited, should be accustomed to apply to their own countrymen, as I feel a perfect conviction, that until the practice gets entirely into the hands of the Chinese, it cannot obtain a permanent footing, nor be of any extensive utility in the empire. Unfortunately, their medical men at present conceive it to be their interest to oppose and calumniate it; and until the suffering of the community, and the private interests of some set of men happen to concur in its behalf, I am afraid that the utmost Europeans can do, will be to preserve it for them until that favourable conjuncture arrives; I will not, however, neglect any feasible opening which may occur of spreading it from this city to the country or provinces. Cochin-China is now the only part of the civilized, I had better perhaps say, cultivated and inhabited globe, to which the practice has not been extended; I have before made the attempt to convey it unsuccessfully, but this season it has been sent in different modes, and with due precautions, under the care of two gentlemen, who are equally competent and desirous to do justice to it; they have also several copies of Sir G. Staunton's version of the treatise which is mentioned in the article of your Journal referred to. I might not, perhaps, have sent you an article of intelligence so much in detail, had I not seen in the report of the success of Mr. Balmis, the Spanish physician, in his practice in South America, and in bringing it to Manilla, a very considerable mis-statement of the circumstances of its introduction here by him. The medical men of Macao had followed it there; several hundred Chinese had submitted to it under my inspection, and the above-mentioned treatise had been published and circulated some months I believe before his arrival, at which time the practice was on a footing, to which he could not give either extension or improvement, at least as far as our countrymen were concerned. His, and some prior attempts at Canton, had been unavailing to give the practice currency, until the return of the British factory to that place.

A. PEARSON.

*Canton, March* 8, 1808.

